# 3D and 4D Printing in the Fight against Breast Cancer

**DOI:** 10.3390/bios12080568

**Published:** 2022-07-26

**Authors:** Sofia Moroni, Luca Casettari, Dimitrios A. Lamprou

**Affiliations:** 1 School of Pharmacy, Queen’s University Belfast, Belfast BT9 7BL, UK; sofia.moroni@qub.ac.uk; 2 Department of Biomolecular Sciences, University of Urbino Carlo Bo, 61029 Urbino, Italy; luca.casettari@uniurb.it

**Keywords:** 3D printing, 4D printing, additive manufacturing, drug delivery, breast cancer

## Abstract

Breast cancer is the second most common cancer worldwide, characterized by a high incidence and mortality rate. Despite the advances achieved in cancer management, improvements in the quality of life of breast cancer survivors are urgent. Moreover, considering the heterogeneity that characterizes tumors and patients, focusing on individuality is fundamental. In this context, 3D printing (3DP) and 4D printing (4DP) techniques allow for a patient-centered approach. At present, 3DP applications against breast cancer are focused on three main aspects: treatment, tissue regeneration, and recovery of the physical appearance. Scaffolds, drug-loaded implants, and prosthetics have been successfully manufactured; however, some challenges must be overcome to shift to clinical practice. The introduction of the fourth dimension has led to an increase in the degree of complexity and customization possibilities. However, 4DP is still in the early stages; thus, research is needed to prove its feasibility in healthcare applications. This review article provides an overview of current approaches for breast cancer management, including standard treatments and breast reconstruction strategies. The benefits and limitations of 3DP and 4DP technologies are discussed, as well as their application in the fight against breast cancer. Future perspectives and challenges are outlined to encourage and promote AM technologies in real-world practice.

## 1. Introduction

Breast cancer is the second most common cancer worldwide, especially among the female population. Male breast cancer is rare, involving only 0.5–1% of new cases [1]. Despite the preventive strategies, such as screening mammography, visualization, and touching the breast, cancer is often diagnosed late, resulting in high mortality. Indeed, according to the World Health Organization (WHO), in 2020 it was estimated that 2.3 million women were diagnosed and around 30% of the cases died [2,3]. Breast cancer is a heterogeneous disease; indeed, the etiology can be considered multifactorial, including both hereditary and acquired factors. The heterogeneity involves patients and tumors, leading to a difference in the prognosis and treatments [4]. The primary risk factors are sex (female) and age (over 40), but also family and medical history and an unhealthy lifestyle (e.g., weight, unbalanced diet, alcohol consumption, smoking), which can increase the incidence [5,6].

**Figure 1:**
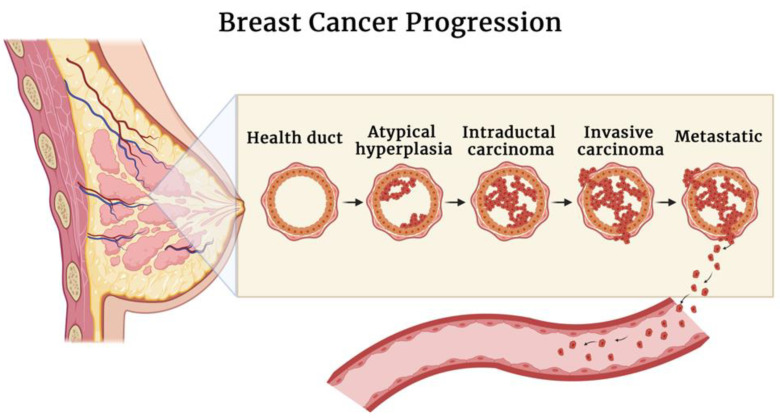
Schematic illustration of breast cancer progression.

Breast cancer is mostly derived from abnormalities in the epithelium (carcinoma), and it encompasses a group of injuries that differ in microscopic aspects and biological behavior. The natural evolvement of breast cancer consists of the progression from non-invasive forms (in situ), in which tumor cells are limited to ducts or lobules, to invasive forms characterized by the spread to the breast stroma and, lastly, to metastatic carcinomas when the tumor spreads to distant sites. Non-invasive carcinoma can be ductal in situ or lobular in situ. Ductal carcinoma in situ (DCIS) is the most common form of non-invasive carcinoma, often associated with recidivism and progression to the invasive form known as invasive ductal carcinoma. Invasive ductal carcinoma, also known as infiltrating ductal carcinoma, is the most common type of breast cancer [7,8,9]. A schematic representation of breast cancer progression is reported in Figure 1.

The growth and development of the mammary gland are dependent on complex interactions between hormones (estrogen and progesterone) and growth factors (human epidermal HER2) with their specific cellular receptors [10]. Estrogen stimulates the normal growth of ducts, while progesterone is responsible for the development of the lobule and alveolar. These lipophilic molecules easily diffuse through the membrane and bind to the receptors present in the cytoplasm that are transported to the nuclei where they interact with the DNA. This interaction will stimulate the genetic response that affects the normal growth of the cell and its function. Thus, knowing the expression of hormones of the tumor is important, as it allows for predicting the prognosis and the possible response to the endocrine treatment and selecting the adequate therapy [6,11].

Conventionally, breast cancer can be classified according to the presence or absence of three biomarkers: estrogen receptor (ER), progesterone receptor (PR), and human epidermal growth factor receptor 2 (HER2), being called hormone receptor-positive or negative. However, the expression of ER, PR, and HER2 receptors is not universal; around 15–20% of breast cancers are hormone receptor-negative, called triple-negative breast cancers (TNBCs). TNBC is an invasive cancer subtype, characterized by aggressive behavior, early relapse, and metastases; in addition, TNBC does not respond to conventional treatments, limiting the survival of patients [12,13].

Breast cancer not only concerns the potential survival of patients but also their well-being, from the aesthetic and emotional point of view. During the last decade, efforts have been made to improve the quality of life of patients and survivors by making progress in the prevention, diagnosis, and treatment of this burden of disease.

This review article encompasses an overview of current strategies in the management of breast cancer. Key aspects and limitations of standard treatments are discussed. In addition, the importance of customizability is underlined. An overview of 3DP and 4DP technologies, materials employed, associated advantages, and challenges is provided. In addition, sections dedicated to recent 3DP and 4DP applications in the fight against breast cancer are reported with regard to treatment and surgery-related approaches. Finally, regulatory aspects and future perspectives are discussed.

## 2. Principles of Therapy for Breast Cancer: Current Treatments and Limitations

The treatment for breast cancer varies according to the clinical and pathological staging. Moreover, considering the options available and the physiological impact of treatments, the selection of the appropriate strategy should include the patient’s wishes (for instance, choosing between breast-conserving surgery or mastectomy) [14]. Indeed, the treatment aims to obtain satisfactory results from the aesthetical point of view, without compromising the control over the tumor or the survival of the patient. Commonly, the initial phase of the treatment involves surgery and radiotherapy; systemic therapies are sometimes necessary, which will be discussed in this section.

### 2.1. Surgery and Radiotherapy

Surgery is performed to resect the primary tumor with the surrounding margin with or without a staging of the axillary lymph nodes. The possibility of performing a satisfactory resection depends on the dimensions and localization of the tumor, the dimensions of the breast, and the necessary margin. Different types of surgery can be performed according to the objective to be achieved. When the tumor is identified, breast conserving surgery or mastectomy can be chosen. In addition, radiotherapy can be necessary to eradicate the residual disease and prevent recidivism [15].

Breast-conserving surgery is the primary treatment, usually associated with radiotherapy. The volume of excised breast and skin should be the minimum possible to preserve normal tissue and shape. Breast-conserving surgery requires a careful assessment of the mammography before the biopsy and the localization of the tumor concerning the margins of the excision. The evaluation of the margin of a sample of excision is fundamental to predicting recurrence and prognosis; however, it can be difficult because an error in the sampling can occur, and there are not standardized methods. If the margins of the excision are clear, the patient can be treated with radiotherapy [16]. Radiotherapy employs high-energy X-rays or gamma rays to kill cancer cells, reduce the risk of local recidivism, and avoid short- and long-term complications. According to the area affected by cancer, radiation can be focused on a specific site or the entire breast. Standard treatments are administered five days a week for a period of six or seven weeks [17]. Alternatively, if margins are positive, mastectomy will be performed. During a mastectomy, the nipple and areola will be removed, significantly impacting patients’ well-being. For this reason, patients are encouraged to undergo to the immediate reconstruction of the removed tissues, although this additional surgery will prolong the hospitalization [16].

#### Breast Reconstruction

Breast reconstruction can be necessary to reshape the breast; the removal of a big tumor can result in breast deformation and dimpling formation, affecting the patient’s wellbeing. The currently available reconstructive technique cannot re-establish the physiological function of the mammary gland but can help breast cancer survivors to restore their body image and thus their self-confidence. Generally, two options are available for the reconstruction: artificial implants or autologous tissue flaps reconstruction; both techniques can sometimes be performed together to achieve better results [18].

Implants are inserted under the pectoralis major muscle or under the breast gland; an expander is sometimes necessary to create the pocket under the muscle and allow skin stretch. They are generally made of an outer shell of silicone and filled with different materials, commonly with silicone gel or sterile salt water. The size, shape, and surface (rough or smooth) can be chosen according to the patient’s needs [19,20]. The employment of implants presents some advantages as the surgery is rapid and the result is aesthetically pleasing. However, downsides are related to possible infections that require the removal of the implant, capsular contracture, implant dislocation, or deformities [21,22]. Otherwise, tissue-based reconstruction can be performed. This procedure consists of rebuilding the breast shape using tissue from the patient’s body, usually from the abdomen, back, or gluteus. The main drawback of this technique is that tissue undergoes natural changes, for instance, weight loss or gain; in addition, the recovery is longer, and the procedure is more complex [23,24]. The final step of reconstruction concerns the nipple–areola area. Reconstruction of this area can be done by surgery or tattooing. However, women that do not wish or are not good candidates to undergo surgery opt for the employment of an external prosthesis that can simulate the natural shape of the breast and/or nipple. The prosthesis can be attached to the bra or can be directly applied to the body through an adhesive band. Usually, external prostheses are made of silicone, and different shapes, sizes, and color tones are available [25,26].

### 2.2. Systemic Therapy

Systemic therapy is often recommended to prevent recidivism and prolong a patient’s survival. It concerns chemotherapy, consisting of a specific cytotoxic compound, and hormonal therapy, based on the modification of the endocrine environment to influence the tumor cells’ growth. Systemic therapy can be administered before the surgery (neoadjuvant chemotherapy) to reduce the size of the tumor or after the surgery (adjuvant chemotherapy) to kill residual cancer cells and prevent recurrences. The potential toxicity of treatments must be considered within the advantages conferred [27,28].

#### 2.2.1. Chemotherapy

Chemotherapeutic drugs interfere with the fundamental cellular processes, provoking cell death. Even if drugs have pleiotropic effects, they can be distinguished according to their primary mechanism of action. Drugs that interfere with the DNA replication act through the alkylation of the base pair of the DNA or by interposing in the double helix of the DNA. Cytotoxicity can be also obtained by altering the integrity of the membrane and impeding the normal homeostasis. For the treatment of breast cancer, commonly alkylating agents, anthracycline (e.g., doxorubicin), antimetabolites (e.g., 5-fluorouracil), and some vinca alkaloids (e.g., vinblastine) are employed. Chemo drugs are often administered in combination as an injection or infusion. The duration of treatment depends on the drug employed and on individual response to it; generally, it ranges from 3 to 6 months in total. Chemotherapy is not specific for tumor cells; the drugs act when cells are in the active phase of the cell cycle. The most common collateral effects are related to the gastrointestinal system (nausea, vomiting, and diarrhea) and hair loss [6,27,28].

#### 2.2.2. Endocrine Therapy

Hormone receptor-positive breast cancer cells are sensitive to hormonal therapy (or endocrine therapy). Therapeutic effects are related to the reduction of the production of estrogen (e.g., ovarian suppression or aromatase inhibitor) or the block of the estrogen effects at a cellular level (e.g., tamoxifen). Ovarian suppression can be achieved by causing temporary menopause using luteinizing hormone-releasing hormone (LHRH) agonist or chemo drugs, or permanently by removing the ovaries (oophorectomy). Another strategy is represented by an aromatase inhibitor, a class of drugs that target the enzyme aromatase, responsible for the production of estrogen. Currently, HER2-2 positive breast cancer can be treated with monoclonal antibodies (e.g., trastuzumab) eventually linked with chemotherapy drugs (e.g., ado-trastuzumab emtansine; the monoclonal antibody allows the target delivery of the chemo drug) or with kinase inhibitors (e.g., lapatinib). Generally, the choice of the treatment varies according to if the woman is pre- or post-menopause. A combination of both types of systemic treatments can be an option, usually for a duration of 5 to 10 years. Side effects associated with endocrine therapy are milder than those of chemotherapy and usually include hot flashes, vaginal dryness, and changes in the menstrual cycle [6,27,29].

However, standard therapies often present limitations such as poor bioavailability, short-term efficacy due to drug resistance, a propensity to relapse, and poor prognosis. In addition, the great variability between individuals and tumors results in a different response to treatment. Therefore, the need for alternative strategies is urgent. Table 1 summarizes the main advantages and disadvantages of conventional therapies.

## 3. Personalized Medicine and Additive Manufacturing

Currently, industry’s pillars are no longer rooted in mass production; they are prioritizing more and more individual needs, marking a new manufacturing era based on demand. Personalized medicine (PM) is a continuously expanding approach that can be applied in the diagnosis, treatment, and prevention of several diseases. Based on individual differences (e.g., biochemical and genomics), lifestyle and environment, and their contribution to the clinical outcomes, PM allows the selection of the ideal clinical path for the specific patient [30,31]. Considering the high heterogeneity of tumors and unique individuality that lead to different treatment responses, PM can represent a promising and beneficial strategy to tailor patient-centric therapies, thus achieving successful results in cancer treatment [32,33]. The realization of PM can be achieved by additive manufacturing (AM) technology [32,34].

**Figure 2:**
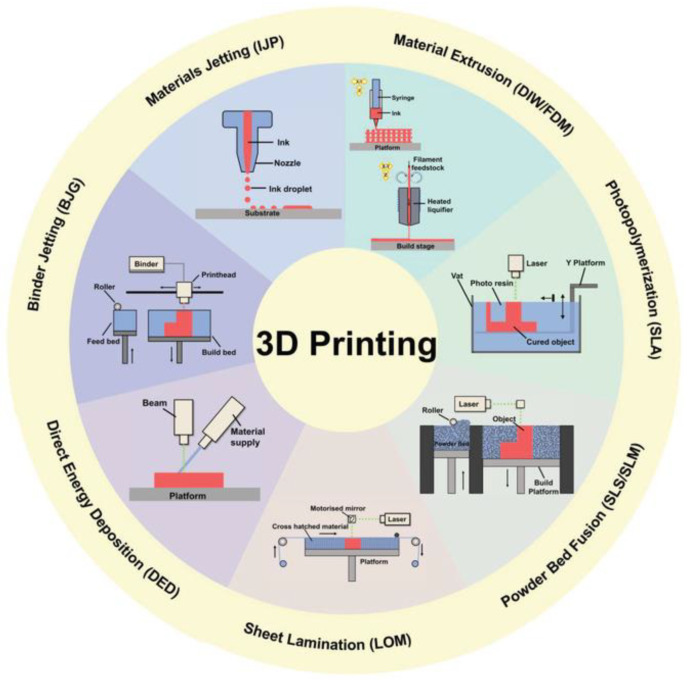
Schematic representation of 3DP techniques, reproduced with permission from Elsevier, license number 5334150461456 [38].

AM encompasses a group of technologies that allow the production of complex objects in a layered fashion using computer-aided control [35]. Three-dimensional printing technologies are classified into seven categories (illustrated in Figure 2): material extrusion, binder jetting, photopolymerization, material jetting, powder bed fusion, sheet lamination, and directed energy deposition [36]. Table 2 reports, for each category, the techniques included, a brief description of the printing process, and their strengths and weaknesses [37].

The possibility of easy customization is one of the key aspects of 3DP; considering pharmaceuticals, it allows the production of a variety of dosage forms, tailored according to the patient’s profile. The personalization of the product may concern the shape, the size, and the drug dose. Moreover, a combination of drugs (e.g., polypills) and drug release rate can be customized, resulting in the reduction of side effects and improvement of the adherence and compliance of the patient [39,40,41].

The continuous evolution of 3DP led to the introduction of a fourth dimension, time.

Tibbits first introduced 4DP in 2013 during his Technology, Entertainment, and Design (TED) Talk conference [42]. This technology can be defined as the manufacturing of dynamic 3D printed objects able to change their morphology and/or characteristics as a function of time. The self-transformation, which should be predictable and programmable, is provided by one or more external stimuli, such as the variation in the pH, temperature, humidity, light, or the presence of a magnetic field. The stimulus is selected according to the final application, and it will affect the material choice. Common transformations are related to shape-shifting abilities, such as folding, bending, twisting, expansion, and shrinkage, while property changes include the color, the stiffness, and the swelling ratio [43,44,45]. This is achieved by using stimuli-responsive materials, also called “smart materials”, which are discussed in Section 4.2. Four-dimensional printing is a booming technology that requires multiple expertise including chemistry, engineering, and mathematical and computational modeling. This advanced research approach is trusted to revolutionize the future of the manufacturing process and daily life of a wide spectrum of fields, including electronics, aerospace, and engineering [46]. Although its application in the biomedical and pharmaceutical fields is still in the early stages, research is highlighting its enormous potential, especially in expanding the possibilities of personalization [47].

The following sections will focus on current trends and future applications of 3DP and 4DP in the diagnosis, treatment, and prevention of breast cancer. 

## 4. Three-Dimensional and Four-Dimensional Printing

### 4.1. Advantages and Disadvantages

Nowadays, 3DP benefits are well established, especially when compared with the traditional manufacturing process. Among many advantages, rapid prototyping, ease of accessibility, structural control, and cost-effectiveness are the most considered. Moreover, from the environmental perspective, 3DP shows eco-friendly features, as it reduces waste production, chemicals are not necessary, and the production process requires low energy. Nevertheless, its real-life application is restrained due to some limitations such as the restricted build size, post-processing, and lack of regulation. Both the advantages and limitations are automatically translated to 4DP [48,49]. The introduction of the fourth dimension has led to a higher level of breakthrough, particularly in terms of product complexity and customization possibilities. The dynamism, combined with the programmed functionalities, is a key advantage of 4DP. However, there are challenges that should be faced, related to materials, technical aspects, and the design process. Since smart materials are the core of 4DP technology, increasing the availability and gaining a better understanding of the impact of the material properties should be prioritized. More attention should be focused on new materials and on improving the biocompatibility and printability of the currently available materials. Expertise in the materials’ properties and their behavior will enable the design process to be more precise, resulting in higher alteration accuracy and advancements in the potential applications. Furthermore, new triggering mechanisms should be explored to produce faster responses and expand their applications. In the case of biomedical research, sensitiveness to specific biological molecules (e.g., the presence of glucose or enzymes) can increase in vivo feasibility [50]. Robust, efficient, and highly sensitive methods need to be developed to improve the manufacturing quality of the printing process of smart materials, together with the development of theoretical models and design methodologies [51,52].

### 4.2. Materials for 3DP and 4DP

The materials used in AM differ in physical and mechanical properties. The selection of the materials varies according to the technique employed and the desired final products. Printing materials can be liquid, paste, powder, or solid sheets. They include polymers, ceramics, metals, resins, and even food [53,54,55]. Table 3 outlines the most common materials employed in breast cancer applications and their main characteristics.

However, most 3DP materials are not applicable for 4DP. Thus, taking inspiration from nature, efforts have been made to fulfill the need [56].

Four-dimensional printing is based on the employment of a so-called smart material or programmed material. The term smart material indicates a material’s adeptness at changing its function and/or shape upon stimulation, as a change in the physiological parameters or external factors. The choice of the appropriate material is one of the main steps when approaching 4DP, together with the choice of the optimal printing technique and the adequate stimulus. Currently, smart materials can be distinguished between polymers, ceramics, alloys, composites, and metals. Moreover, they can be classified according to the endowed properties, including self-repairing, shape-changing, self-sensing, or self-assembling [57,58]. Smart materials can undergo single or multiple changes, that can be programmed as one-way or two-way. One-way transformations are planned to retain the triggered alteration while the two-way transformations are reversible. Furthermore, changes can be repeatable. Nevertheless, the range of available materials needs to be expanded in order to increase the implementation of 4DP; thus, research is required to focus on the compatibility and design of precise responsive materials [51].

Herein, the focus will be on shape-changing materials, specifically shape memory materials and smart hydrogels. 

#### 4.2.1. Shape Memory Materials

The response to a specific trigger can be expressed through several transformations; among these, shape shifting has attracted great attention. When considering shape shifting, it is important to distinguish between a shape-changing effect and shape memory effect. Indeed, the first case describes the ability to deform to a temporary shape that is immediately restored when the driving stimulus is removed. On the contrary, the shape memory effect defines the ability to deform to a temporary shape that is maintained over time and can recover back to the original form in response to specific external stimulation. Thus, the shape memory effect can be described by two factors: the shape fixity ratio (ability to maintain the temporary shape) and the shape recovery ratio (efficiency in recovering the original shape). Furthermore, multiple temporary shapes can be sequentially acquired [43].

Different shape memory materials have been developed: a brief description of alloys and polymers is provided in the following sections. 

##### Shape Memory Alloys

**Figure 3:**
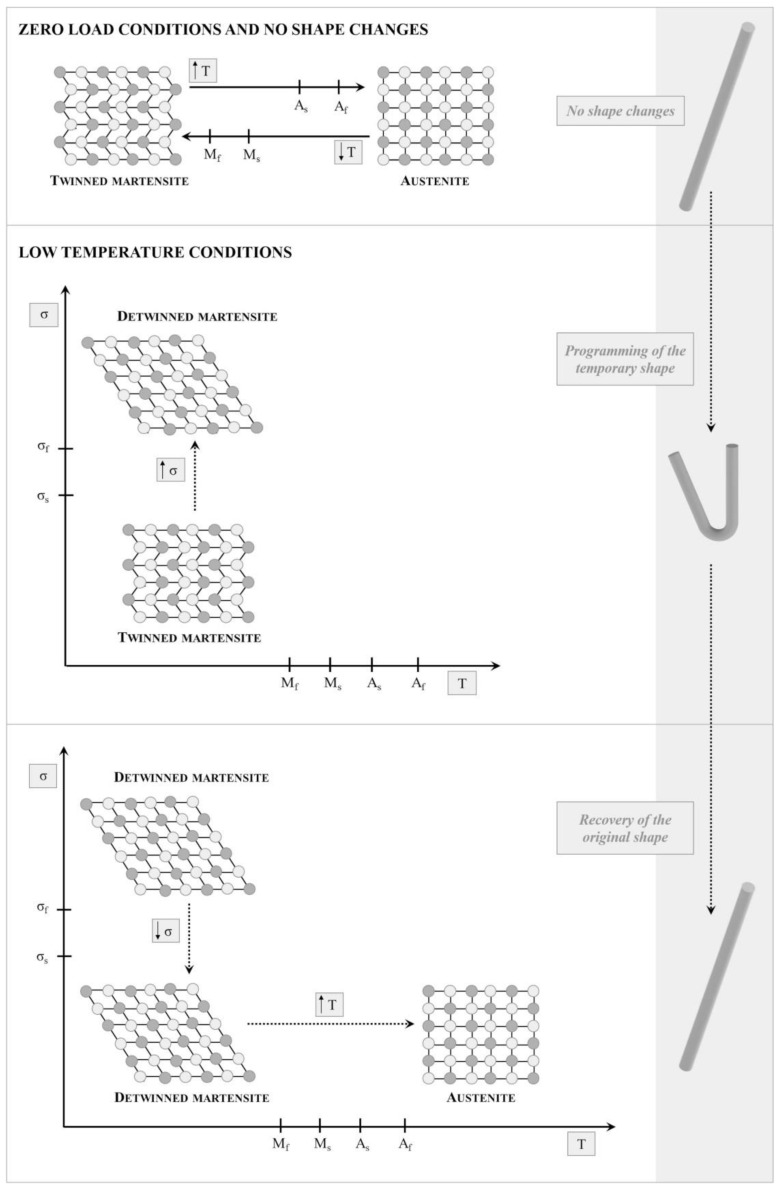
Shape memory effect of alloys, reproduced with permission from Elsevier, license number 5334150011086 [62].

The shape memory effect for alloys is based on the reversible transformation between two crystalline phases: austenite and martensite, each of which is characterized by distinctive characteristics. The austenitic phase presents a cubic structure; while the martensitic structure varies according to the alloy composition (e.g., tetragonal, orthorhombic, or monoclinic structure). Moreover, different martensitic orientations can be observed, which are called variants. If one variant prevails, the martensite is called “detwinned”, whereas if combinations of variants coexist, it will be called twinned martensite. The shape memory effect is mainly activated by heat. The austenitic phase represents the initial phase that is subject to deformation upon cooling at the martensitic start temperature (Ms) and will be fully transformed to twinned martensite at the martensitic finish temperature (Mf). The recovery to austenite is initiated at the austenitic start temperature (As) and will take place at the austenitic finish temperature (Af). Moreover, when sufficient stress is applied to the martensitic phase, the detwinning process can occur, resulting in temporary deformation of the orientations (Figure 3). The detwinned orientation can be preserved even when the stress is released. Currently, the most investigated alloys are copper, nitinol-titanium (NiTi), iron, and their combination with other materials. Among them, NiTi systems are the most explored, because of the better biocompatibility, good processability, and good properties, such as the high actuation stress and recoverable strain. However, due to their complex production, high cost, potential toxicity, and limited recovery, other materials are preferred over alloys [59,60,61].

##### Shape Memory Polymers (SMPs)

**Figure 4:**
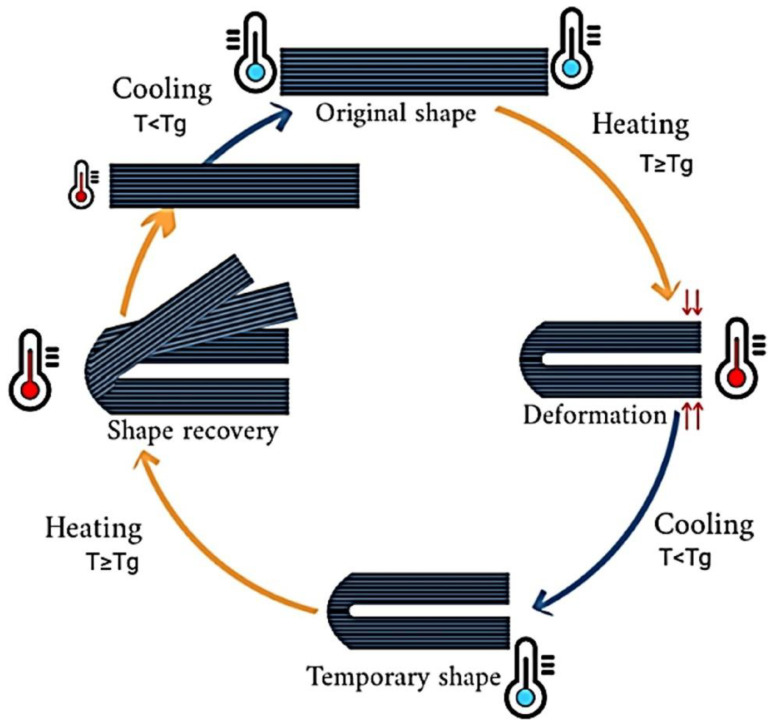
Shape memory effect of polymersreproduced with permission from Elsevier, license number 5334150884441 [68].

SMPs are wildly employed for 4DP applications because of their favorable properties, including their low cost, light weight, good manufacturability, high flexibility, and deformation ability. When the transformation is induced by heat, materials are called thermally responsive. Commonly, the shape transformation occurs by heating above the transition temperature at which the polymer chains can easily move due to the increase in entropy. By applying an external force, the printed object shifts to a temporary shape, characterized by lower entropy, thus reducing mobility. The rearrangement is fixed by cooling below the transition temperature. When removing the external force, the temporary shape is retained until heating again above the transition temperature, at which point the original shape will be spontaneously recovered. This programming process can be repeated, and different temporary shapes can be obtained (Figure 4) [58,62]. In the biomedical field, thermo-responsive polymers are the most investigated materials. Among them, PLA showed to be a promising candidate for 4DP applications, in particular to produce vascular stents [63,64] and bone tissue scaffolds [65,66], allowing minimal invasive surgery. However, some drawbacks limit the use of polymers in 4DP, for instance, the strength and stiffness [67]. For this reason, combinations of materials can sometimes represent an opportunity to improve materials’ properties; alternatively, newly synthesized materials should be investigated. 

#### 4.2.2. Smart Hydrogels

Hydrogels are hydrophilic materials that, according to their origin, can be synthetic or derived from nature. Natural hydrogels comprise polysaccharides, such as alginate, chitosan, hyaluronic acid, and cellulose, and proteins, such as gelatin and collagen. Synthetic hydrogels can be a homopolymer (one monomer); copolymer (made of two co-monomer units); or multipolymer (composed of three or more co-monomer units). Among the synthetic polymers, polyacrylamide, polyethylene glycol (PEG), and acryl acid are the most common [69,70]. Hydrogels are suitable for 4DP purposes, especially for the shape-morphing transition, because of their intrinsic swelling ability. Indeed, when exposed to a stimulus, they can undergo volume modifications, such as swelling and shrinkage, or structural modifications such as sol/gel transition [71]. This change can be exploited for drug delivery purposes. Recently, Zu et al. developed a poly(N-isopropylacrylamide) (PNIPAM)-based capsule shell for smart controlled drug release. The presence of PNIPAM introduced temperature responsiveness; thus, the controlled drug release can be attributed to the modification of the internal pore size, achieved by the variation of the temperature above or below the lower critical solution temperature (LCST) [72]. However, hydrogels show some limitations in terms of mechanical strength, stability, and manipulation possibilities. For this reason, they are often crosslinked with polymers. In 4DP, the printed object can be completely responsive, or the structure can comprise active and passive components. Commonly, hydrogels are crosslinked with non-responsive polymers in order to create a layered platform: the hydrogel will form the active layers, able to absorb water and swell, while the passive layers will be made of the polymer. The deformation is adjusted by varying the distribution of the two layers. This mechanism is commonly used to create self-folding devices. However, the deformation is not defined and the response requires time [49,58].

## 5. Three-Dimensional Printing in the Fight against Breast Cancer

Three-dimensional printing has shown a significant impact in revolutionizing the standard conception of illness and treatment. In breast cancer, its potential clinical significance encompasses all aspects, including the diagnosis, treatment, and aesthetic outcome. The following sections will provide an overview of current applications of 3DP in the fight against breast cancer. Despite some tools to diagnose breast cancer that have been developed [73,74,75], this review will focus on treatment and surgery-related applications.

### 5.1. Three-Dimensional-Printed Prototypes

**Figure 5:**
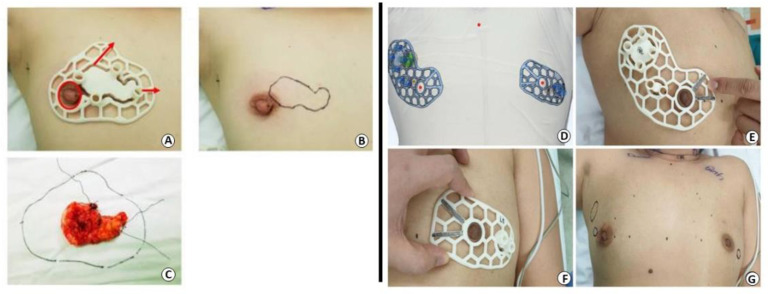
Patient-specific surgical guides [80,81].

Breast and tumor prototypes are key instruments used for different purposes, including diagnostic tasks, surgery training, preoperative planning, treatment optimization, and drug screening. Three-dimensional-printed physical phantoms are employed to mimic the patient’s anatomy, both from the external and internal perspectives, allowing the evaluation and optimization of imaging techniques [76,77]. In addition, they can be useful to predict the dose distribution for safe and effective radiotherapy treatments [78]. Three-dimensional-printed models can additionally help surgeons to plan and make decisions, as well as be employed for training activities or to increase the efficacy of patient communication [79]. From a practical perspective, 3DP tumor models were developed to localize the tumor, providing a physical model of the area in order to facilitate and guide the resection. For this purpose, breast images are fundamental to acquiring necessary information enabling the production of an accurate design. Wu et al. reported the application of MRI-based patient-specific 3DP surgical guides (Figure 5) for breast-conserving surgery in patients with ductal carcinoma in situ and invasive ductal carcinoma. In both case studies, the results suggested that surgery was successfully performed with clear margins [80,81].

### 5.2. Three-Dimensional Printing Application in the Treatment of Breast Cancer

#### Drug-Loaded Implants

As previously discussed, traditional treatments for breast cancer include surgery, radiotherapy, and systemic therapy. However, these strategies have limitations, underlining the need for alternative therapies. A drug-loaded implant can represent a promising option to efficiently control local drug delivery and reduce side effects. Indeed, implants allow the reduction of dose and frequency of drug administration, resulting in minimal systemic toxicity. Moreover, multiple drugs can be loaded into the same device, enabling a synergic effect. Implants can be inserted near the tumor, to deliver chemotherapy drugs, or after the surgery resection to prevent recurrence and metastasis. Three-dimensional-printed implants for breast cancer treatment can be designed in a variety of geometries using different materials and drugs. For instance, Dang et al. produced a porous PCL implant loaded with doxorubicin (DOX) [82], while Fan et al. reported the manufacturing of an ultrahigh-molecular-weight-polyethylene scaffold loaded with 5-fluorouracil (5-FU) using the FDM technique [83]. Quiao et al. developed, by material jetting, poly-lactic-co-glycolic acid (PLGA)-based implants to simultaneously deliver the combination of drugs in a controlled manner. In an earlier study, they investigated the effect of DOX and cisplatin, and later that of 5-FU and NVP-BEZ235. In vitro and in vivo studies showed promising results in suppressing tumor growth, suggesting the benefit of these drug delivery systems [84,85]. PLGA was also employed by Shi et al. to manufacture, through material jetting, a multifunctional device with the aim to inhibit cancer growth and provide wound healing. For this purpose, DOX and 5-FU were chosen as therapeutic compounds, while gelatin crosslinked with chitosan was added to promote wound healing and tissue regeneration and to confer pH responsiveness. In vitro and in vivo studies confirmed the hemostasis ability, blood absorption, and wound healing ability. In addition, the controlled delivery of drugs was achieved [86]. Multipurpose implants can promote anticancer activity and, simultaneously, tissue repair as additionally suggested by Luo et al. Moreover, they investigated the possibility of monitoring the implant performance during in vivo imaging by incorporating Mn^2+^ ions and polydopamine (PDA) to the bioprinted device [87]. Furthermore, 3DP technology offers the opportunity to produce advanced devices: He et al. combined a 3DP bio-glass scaffold with an immune adjuvant to treat bone metastasis of breast cancer. The device consisted of the incorporation into the scaffold of a niobium carbide (Nb2C) MXene nanosheet coated with mesoporous silica, loaded with R837 (an immune adjuvant). The resulting product possessed photothermal and immune activation properties, able to attack the tumor and avoid metastasis and recurrences. In addition, the implant supported osteogenesis. Overall, the authors believe that their study could represent a promising strategy to produce in situ tumor vaccines [88]. 

### 5.3. Three-Dimensional Printing Application in Breast Reconstruction

#### 5.3.1. Scaffold-Guided Reconstruction

**Figure 6:**
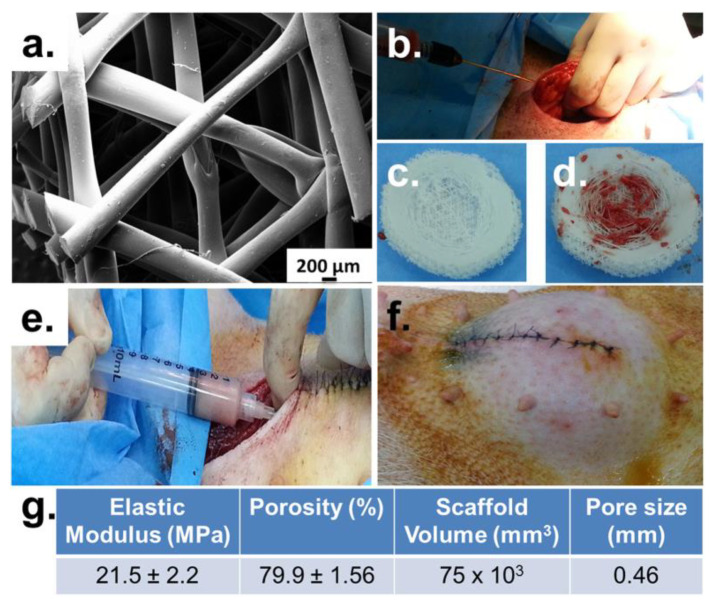
Showing (**a**) structure of the scaffold; (**b**–**f**) implantation of the scaffold and fat injection process; and (**g**) scaffold’s properties [94].

Nowadays, a variety of reconstructive strategies are available, with the aim to re-establish the self-confidence of patients post-mastectomy. One strategy is represented by the development of a 3D scaffold able to guide cellular interaction and tissue formation. Ideally, the scaffold should match the natural mechanical properties of the breast, allow tissue regeneration, and maintain a pleasing cosmetic shape of the breast. An additional feature is represented by the possibility to stimulate the adipose tissue by incorporating adipose-derived stem cells (ADSCs) into the device. The design of the product is tailored according to images captured by computed tomography or magnetic resonance, in order to meet the patient’s needs [89,90,91,92,93]. Among the materials suitable to produce 3DP scaffolds, PCL has been largely employed owing to its beneficial properties, such as biocompatibility, good processability, and good mechanical properties. In 2016, Chhaya et al. implanted a PCL-made scaffold in minipigs and compared the effect of the empty scaffold, the scaffold loaded with lipoaspirate, and the scaffold in which the autologous fat was injected after 14 days of implantation. After 24 weeks, angiogenesis and adipose tissue regeneration were observed, in particular for the device with delayed injection, suggesting the potentiality of this technique (Figure 6) [94]. With the aim to better control fat distribution, in 2019, the same group developed, by FDM, a complex design composed of independent internal and external structures. The geometry of the internal structure was designed to guide tissue regeneration, being modified according to the patient’s profile, while the external structure provided biomechanical support [95]. The potentiality of 3DP scaffolds in fat grafting was additionally suggested by Bao et al. Nonetheless, they underlined the need for optimization related to the ability to efficiently provide vascularization and to the long degradation profile of the product [96]. Thus, to improve the in vivo performance of 3DP scaffolds, different aspects should be considered. Zhou et al. emphasized the importance of the design on the mechanical properties of the final product, fundamental to mimicking the native breast characteristics [97]. Given the importance of resembling the natural breast properties, Tytgat et al. focused on the development of soft scaffolds, using a material extrusion-based 3D printer. Studies confirmed similarities between the manufactured devices and native tissue. Moreover, scaffolds were able to support ADSCs differentiation [98]. As previously mentioned, one of the main advantages of the 3DP technique is its versatility. This advantage was exploited by Dang et al., who combined breast regeneration with the possibility of simultaneously administering the drug to prevent surgical complications. Indeed, in the proof-of-concept study, they developed by FDM a PCL scaffold able to guide tissue regeneration. The scaffold was subsequently loaded with chemotherapeutic drugs (DOX and paclitaxel) and antibiotics (cefazolin) to prevent recurrence and infection [99]. In 2017, two start-ups were launched, aiming to provide a valid alternative to silicone prostheses. LATTICE MEDICAL developed a bioprosthesis, called MAT(T)ISSE, using the FDM technique. MAT(T)ISSE provides volume to the breast and allows adipose tissue regeneration. In addition, it degrades in one year, requiring only one surgery [100]. The company BELLASENO manufactured a PCL resorbable scaffold that completely degrades in 2–3 years. The product, called SENELLA BREAST, is designed to be employed for breast reconstruction and augmentation, and it allows fat injection [101]. 

#### 5.3.2. External Prostheses

For breast cancer survivors who have undergone a mastectomy, breast reconstruction is not always possible; therefore, an external prosthesis can be an alternative for the patient’s wellbeing. Sometimes, however, standard prostheses are not able to meet the requirements of every individual, resulting in general dissatisfaction. The main problems encountered are related to failure to appear realistic due to the tactile feel, excessive weight that can lead to balance and posture issues, and lack of adaptation to the breast anatomy and natural movements [102,103,104]. To achieve higher satisfaction, increased customization is necessary [105]. The first step in the manufacturing process of prosthetics involves the scan of the anatomy of the patients, which is essential to obtain accurate information. From the acquired information, the prosthesis can be designed accordingly and finally produced either by direct printing or by printing the mold that will be subsequently filled [106]. This strategy was employed by Maillo et al., who developed an external prosthesis in thermoplastic polyurethane (TPU) and polyvinyl acetate (PVA) using the FDM technique [107]. Alternatively, Unit et al. produced a mold to be filled with silicone, using the SLS technique [108]. Three-dimensional-printed molds can also be a useful template to help the surgeon to shape the flap that will be employed in the autologous reconstruction of the breast. This procedure reduces the duration of the surgery and allows for a better outcome, in particular for the symmetry of the breast [109,110]. An interesting study was conducted by Hao et al., who explored the possibility of combining breast reconstruction and chemotherapy treatment by producing a local implant with polydimethylsiloxane loaded with paclitaxel and DOX microspheres. For this purpose, the implant mold was designed and produced by 3DP to confer customizability. In vivo studies conducted on mice suggested that the system could prevent recurrence and metastasis formation in mice [111].

#### 5.3.3. Nipple–Areola Complex Reconstruction

The last step of breast reconstruction concerns the nipple–areola complex, whose restoration significantly impacts the overall satisfaction of the patient. At present, the application of 3DP technology in this area is limited to the production of acellular scaffolds or bio-printable ink loaded with cells, able to support and guide the natural tissue restoration [112,113]. Aiming to avoid possible rejection and achieve a long-term nipple projection, Samadi et al. manufactured a cylinder scaffold made of polylactic acid (PLA) embedded with autologous tissue from the costal cartilage. Despite the promising results in mice, the authors found some limitations, mainly related to the degradation time and the rigidity of the scaffold matrix [114]. Therefore, in a later study conducted by the same group, the PLA was replaced with poly-4-hydroxybutyrate to produce a fully absorbable device [115]. Healshape is a start-up founded in Lyon in 2020. They are developing bioprinted prostheses both for breast augmentation and for nipple–areola complex regeneration. The design allows cell colonization and tissue regeneration [116].

Although 3DP technology provides numerous advantages in the manufacture of advanced and complex devices at lower costs, the clinical applications are still limited. Therefore, further improvements should be achieved to make it more accessible in practical employment. 

## 6. Four-Dimensional Printing Applications in Breast Cancer

**Figure 7:**
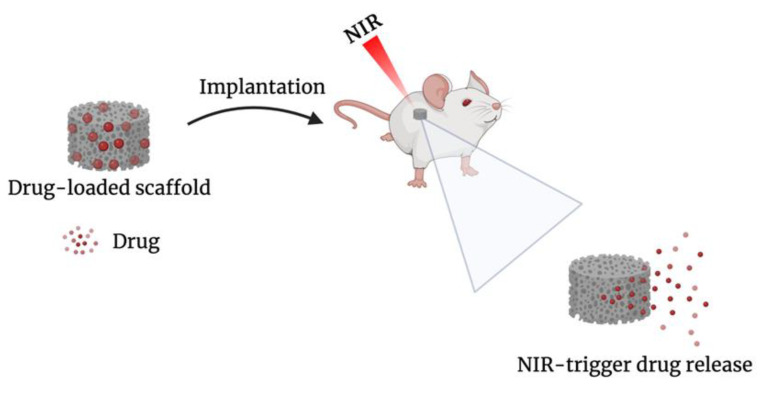
Schematic illustration of NIR-triggered drug release.

Despite the promising advantages that 4DP enables, the field is still in its infancy. At present, the only documented application of 4DP in breast cancer consists of the development of scaffolds with NIR-triggered DOX delivery (Figure 7), using extrusion-based 3DP. In 2020, Wei et al. developed a core–shell scaffold. The incorporation of PDA provides the responsiveness to NIR irradiation; indeed, under stimulation, the core underwent sol/gel transition, resulting in drug release. The resulting scaffold could potentially be placed after breast conservative surgery; moreover, the irradiation provided a photothermal effect that could prevent recurrence [117]. Later, based on the same principle, the research group produced a drug-loaded alginate–gelatin core scaffold coated with PCL and subsequently coated with PDA. The presence of PCL enabled a controlled and sustained release of the drug. Moreover, the scaffold showed wound healing properties [118]. NIR-triggered drug release represents one of the multiple opportunities that 4DP could provide in breast cancer management. Given the benefits of 4DP, especially in the personalization possibilities, other strategies should be explored. Considering the recent results achieved by shape memory effect materials, they are expected to provide a valuable contribution in the pharmaceutical field, including in breast cancer treatment and reconstruction purposes. 

## 7. Regulatory Considerations

Currently, the major obstacle that limits the application of AM in real-life practice is the lack of well-defined regulatory guidance. Indeed, 3D-printed products are regulated under the same pathways as non-additive manufactured devices. They are classified as Class I, II, or III, depending on the risk of injury for the user and the level of control necessary to guarantee safety and effectiveness [119]. In 2017, the Food and Drug Administration’s (FDA) Center for Devices and Radiological Health (CDRH) published a guideline on 3D-printed medical devices and prosthetics. The guideline covers three main aspects: the design, manufacturing, and tests to be performed. In the Design and Manufacturing section, technical considerations are included to fulfill the Quality System (QS) requirements to ensure product quality and safety. While the Device Testing Considerations section provides the information requested for the submission and approval [120]. However, guidelines can be restrictive and involve a long bureaucratic process. Scaling up to comply with the Good Manufacturing Practice (GMP) can require high costs. Moreover, meeting the FDA requirements for clinical trials can be difficult considering the nature of AM, which is based on individuality [121]. From this, it follows that traditional approaches are preferred. To take a step toward the implementation of AM in everyday life, collaborations within academia, industry, and FDA are fundamental. 

## 8. Conclusions and Future Perspectives

During the past years, advancements in AM technology and material science, combined with the increasing interest in PM, have prompted new strategies in the biomedical field. Three-dimensional printing and four-dimensional printing technologies have shown the potential to revolutionize cancer management by providing a higher level of personalization. Indeed, tumor heterogeneity restricts the efficacy of standard treatments, highlighting the urgency for alternative approaches. PM is focused on the impact of different factors (such as lifestyle and environmental conditions) on patients’ responses and clinical outcomes. Based on these differences, tailor-made strategies can be developed. At present, the main application of AM in breast cancer concerns the development of drug-loaded implants and scaffolds. Ideally, the devices should provide controlled drug delivery, assist tissue reconstruction, and resemble natural tissue. However, sometimes the immediate aesthetical outcome is overshadowed. Considering the high emotional impact on breast cancer survivors, research should be addressed to provide complete satisfaction to patients, considering both the treatment and aesthetic success. The possibility of theragnostic devices should also be explored to confer an additional tool over the tumor control. Although 4DP has provided a new strategy to achieve a complex and accurate design with high customizability, research is still in the initial stage. Challenges related to technological and material aspects require in depth-understanding to achieve significant advancements and expand the application possibilities. Moreover, understanding the complexity and dynamism of biological systems is a key requirement. In addition, quality aspects and regulatory approvals should be further discussed to successfully translate research into clinical practice. Once the challenges are overcome, AM will provide endless possibilities and breakthroughs in the fight against burden diseases, including breast cancer. 

## Figures and Tables

**Table 1:** Advantages and disadvantages of current standard therapies for the treatment of breast cancer.

	**Treatment**	**Advantages**	**Disadvantages**
*Surgery*	**Breast conserving**	Not invasive, preserves the natural shape of the breast.	Possibility of recurrence.
	**Mastectomy**	Lowers the chance of recurrence.	Invasive, poor cosmetic outcome, high emotional impact.
*Systemic Therapy*	**Chemotherapy**	Before surgery facilitates the removal of the tumor and enables a less invasive procedure, lower chance of metastasis and recurrence.	Unpleasant side effects (e.g., nausea, fatigue, hair loss).
	**Endocrine Therapy**	Milder side effects than chemotherapy, possibility to combine it with chemotherapy or radiotherapy to achieve better results.	Limited to hormone receptor-positive breast cancer, higher possibility of resistance to treatment, early menopause.

**Table 2:** Three-dimensional printing techniques, brief description of the printing process, strengths, and weaknesses.

Category	Technique	Brief Description	Strengths	Weaknesses
**Material Extrusion**	Fused deposition modeling (FDM); direct ink writing (DIW)	Thermoplastic materials or semi-solid inks are extruded through a nozzle.	Good variety of materials, low costs.	Low resolution and time consuming.
**Binder Jetting**		The build material, in the form of powder, and the binder material, generally liquid, are alternatively deposited into the printing bed.	Scalability, high speed.	Poor accuracy, post processing.
**Photopolymerization**	Stereolithography (SLA); digital light processing (DLP)	The final object is obtained through a chemical reaction (photopolymerization) triggered by irradiation.	High accuracy, high speed.	Limited material availability (photo-resins), high costs, post processing.
**Material Jetting**		Material is deposited dropwise or continuously onto the printing bed through a printing head.	Low cost, high speed, scalability.	Limited material availability (polymers or waxes), support necessary.
**Powder Bed Fusion**	Direct metal laser sintering (DMLS); selective laser melting (SLM); electron beam melting (EBM); selective heat sintering (SHS); selective laser sintering (SLS)	Laser or electron beams are applied as thermal source to melt powder particles and build the device.	Good resolution, wide range of materials, complexity of the design achieved.	Small product size, high cost, time consuming.
**Sheet Lamination**	Ultrasonic additive manufacturing (UAM); laminated object manufacturing (LOM)	The material, in the form of sheets, is cut by a laser according to the desired design. Each layer is bonded by pressure, temperature, or adhesive coating.	Low costs, robust.	Low resolution and poor accuracy, post processing.
**Direct Energy Deposition**	Direct light fabrication (DLF); laser engineered net shaping (LENS); direct metal deposition (DMD)	Powder or wire material and the substrate are simultaneously melted using an energy source (laser or electron beam). Firstly, the substrate will create the melt pool where the material will be deposited.	Production of dense part with microstructures, ability to control the structure.	Post-processing, time consuming, low material availability.

**Table 3:** Most common AM materials applied for breast cancer and their main characteristics.

Material	Characteristics	Breast Cancer Application
**Polycaprolactone** (**PCL**)	Low melting temperature, slow degradation rate, good mechanical properties.	Scaffold to guide breast reconstruction and drug delivery.
**Polylactic acid** (**PLA**)	flexible, slow degradation rate.	Nipple–areola complex scaffold.
**Poly**(**lactic-co-glycolic acid**) (**PLGA**)	High degradation rate. High mechanical strength, good processability, high melting temperature.	Scaffold for drug delivery.
